# Stafne bone cavity containing ectopic parotid gland^☆^

**DOI:** 10.1016/j.bjorl.2016.02.004

**Published:** 2016-04-14

**Authors:** Mesut Kaya, Kadriye Serife Ugur, Elif Dagli, Hanifi Kurtaran, Mehmet Gunduz

**Affiliations:** Turgut Ozal University, Faculty of Medicine, Department of Otolaryngology Head & Neck Surgery, Ankara, Turkey

## Introduction

In 1942, Stafne^1^ described 35 asymptomatic cases of a cortical depression of the lingual side of mandible with unilateral radiolucent cavities located in the posterior region of the mandible, between the third molar and mandibular angle, below the inferior mandibular canal and above the mandibular base. He reported the cases as “bone cavities situated near the angle of the mandible”. These lesions are defined as pseudocysts without an epithelial lining. Although the majority of these cysts are seen in the jaw angle below the mandibular nerve, they are also diagnosed in other locations such as the anterior mandible. As its controversial pathogenesis explanations, this lesion has been called of many names including static bone cavity or defect, latent or idiopathic bone cavity, ectopic submandibular or sublingual gland in the mandible, lingual cortical mandibular defect or depression, developmental bone defect of the mandible and Stafne cyst, defect or cavity.

Considering most of the cases, Stafne cavity is diagnosed incidentally in routine radiologic examinations in asymptomatic patients. In panoramic radiograms it appears to be a dense circumscribed radiolucency in mandibular angle, below mandibular canal, mostly in one focus. Stafne cysts are considered stable structural changes of the mandible and do not require surgical intervention. But in literature, there are reports of patients operated for this entity, with descriptions of cavities containing submandibular gland tissue, muscles, fibrous connective tissue, blood vessels, fat or lymphoid tissue.^2^ In this particular paper we present a Stafne bone cavity containing ectopic parotid gland.

## Case report

**Figure 1:**
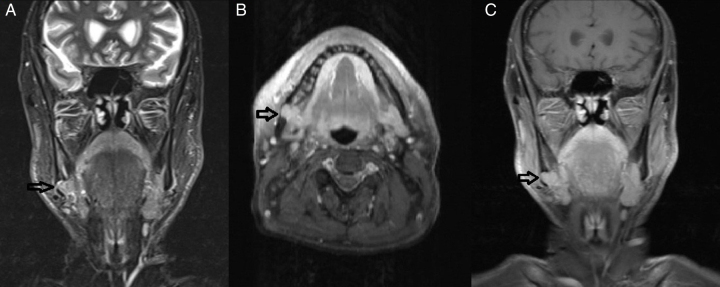
The magnetic resonance images of the lobulated mass of right submandibular region (black arrows) in T1A sections (A), and in T2 fat suppressed sections (B and C).

A 60-year-old male patient with complaint of pain in right side of floor of mouth, was referred to our otolaryngology clinic of a tertiary academic center, with a MRI study showing a solid mass of the right submandibular region diverging from surrounding structures, such as submandibular gland and muscles with regular borders. The lobulated mass showed diffuse contrast retention and intermediate density in T1A sections, hyperintensity in T2 images and did not suppressed in fat suppressed series. Also this mass was filling the space situated anteriorly to the right mandibular angle, formed as a well-bordered cortical depression with an intact inferior mandibular line (Fig. 1).

**Figure 2:**
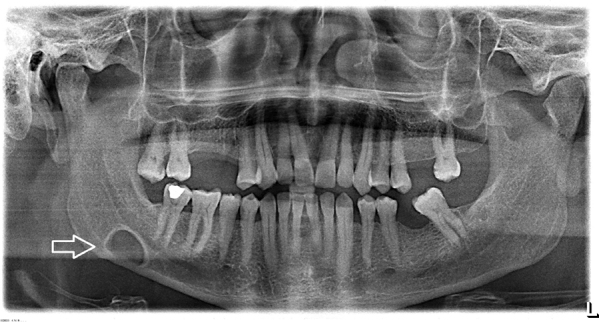
Oval shaped, well-bordered cystic lesion (white arrow) between second molar and the mandibular angle, slightly above the inferior mandibular line in panoramic radiograph.

The patient was having pain for 3 months and antibiotics and anti-inflammatory agents had been used, with no pain relief. A panoramic radiography demonstrated an oval shaped and well-bordered cystic lesion between the second molar and the mandibular angle, slightly above the inferior mandibular line. The third molar had been extracted many years ago, and the cyst was not involving the second molar roots. Surrounding borders of the cyst were more dense (Fig. 2). Head and neck examination revealed no pathologic finding and no pain at palpation of the neck.

**Figure 3:**
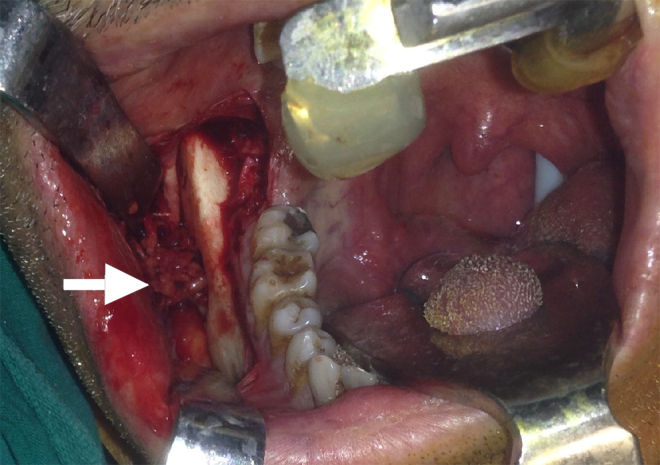
Intraoperative photo of lobulated mass (white arrow) presented through the window opened lateral mandibular wall of Stafne cyst.

An informed consent was obtained and surgical treatment took place. Intraoral incision was performed on the buccal mucosa near the gingivobuccal line. Periosteum of mandible was lifted trough the inferior mandibular line and a window was opened from the buccal side of mandible using a burr. The mass could be observed just behind the thin cortical outer plate and the cavity did not have any epithelial lining. It was non-capsulated, solid, fragile and easily detachable from the submandibular gland and muscles, but strictly attached inferiorly to lingual nerve (Fig. 3). The mass was removed without complication.

**Figure 4:**
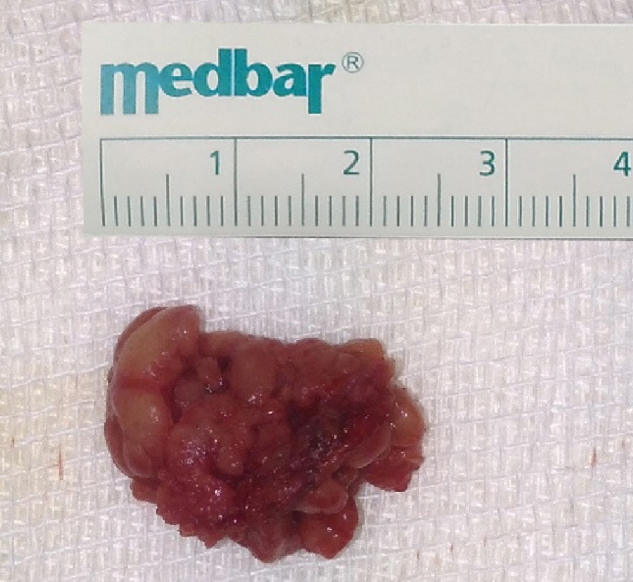
Macroscopic image of ectopic parotid tissue.

**Figure 5:**
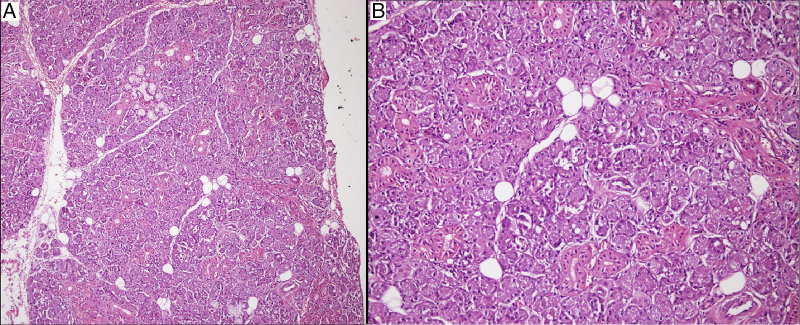
In hematoxylin-eosin staining, the mass was consisted of pure serous glandular tissue in 100× magnification (A). Acinar cells contained zymogen granules and ductal system was intact in 200× magnification (B).

The mass was glandular like shape macroscopically (Fig. 4). Histopathologic findings showed the presence of glandular tissue and ectopic parotid gland (Fig. 5). Acinar cells contained zymogen granules and ductal system was intact. Thereafter we again looked at MRI images to verify if both parotid glands were present in their usual locations.

## Discussion

Accessory parotid gland is a glandular tissue located on the masseter muscle, lying closely or craniolateral to the Stensen's duct, but separated from anterior border of the parotid gland. Accessory parotid gland has a specific ductal system which drains into the Stensen's duct.^3^ Presence of parotid gland tissue outside the natural place or accessory location is called ectopia and can be seen with or without the absence of the parotid gland at its normal location. In the embryo, between sixth and seventh week, major salivary glands start to develop as ectodermal invaginations in the floor of the branchial part of the primitive mouth for parotid gland, and a formation of endodermal invaginations for submandibular and sublingual glands. These cells migrate into the underlying mesenchyme starting their opening of the future excretory duct. During embryogenesis, all salivary glands show close interactions with adjacent lymphatic tissue which explain why ectopic salivary gland tissue most commonly located in the cervical lymph nodes.^4^ In the literature very rare locations of ectopic salivary glands have been reported, including pineal gland, thyroglossal duct, thyroid gland, parathyroid gland, pharyngeal tonsil, lingual tonsil, rectum, stomach and vulva. Although salivary gland heterotopia in various organs is observed, well organized salivary glandular tissue, or a well structured ectopic parotid gland are very rare conditions.^5^ Developmental abnormalities of the branchial apparatus end up with malformations like sinuses, fistulas or cysts. Youngs and Schofield^6^ suggested that ectopia of salivary gland is a result of an anusual persistence and differentiation of the endodermal remnants of the pre-cervical His sinus.

Ectopic salivary glands mostly arise from the anterior triangle along the medial edge of sternocleidomastoid muscle and the most common location is the base of the neck.^7^ Usually ectopic salivary glands present as sinuses opening externally and secreting a clear liquid like saliva especially during eating. Bilateral presentation of ectopic salivary gland is rare.

Barbuscia et al.^8^ described ectopic parotid gland of the middle neck, structured as normal parotid parenchyma with multi-lobular appearance. Histological examination which is most similar to our case, revealed, acinar glands with serous secretions containing abundant zymogen granules in its compounds, completely preserved.

Our case of ectopic parotid gland was located in Stafne's cavity. When Stafne^1^ introduced this entity, he advocated the developmental theory, saying that it could occur due to a deficiency in ossification in the area filled by Meckel's cartilage. However, it seems unlikely because no presentation under 11 age has been reported, and peak of incidence is usually around the fifth and sixth decades. A widely accepted theory is that the mandibular defect occurs due to pressure atrophy caused by the submandibular gland, since postoperative findings have confirmed the presence of submandibular salivary gland in the cavity.^9^ Quesada-Gómez et al., noted a compensatory hypertrophy and fibrosis caused by lymphocytic infiltration and reduced secreting and increasing in size with age. Minowa et al. showed abnormal vasculature in cavity both radiologic imaging and histopathologically rather than salivary glands, and suggested that vascular lesion could be another explanation for etiology of Stafne cyst. It was proposed that the recanalization of thrombosis, and occlusion of branches of the facial artery could become tortuous because of arteriosclerosis and hypertension which increase with age, consistent with the peak of incidence. Although the submandibular gland can weaken arterial pulses, the mandible is subjected to pressure from the arterial pulses. The pressure on the mandible is thought to be the cause of the bone cavity.

Content of the pseudocyst is usually inflamed salivary gland which is the most common finding in operated or imaging studies of Stafne cavity. Muscles, fibrous tissue, fat, lymphoid tissue, vascular structures or devoid of content are reported in a minority of cases.^9^ Considering the mobility of the mouth floor and neck, unintentional displacement of adjacent tissues during surgical manipulation or intermittent gland repositioning may occur. Our case supports the glandular pressure theory with ectopic parotid gland.

The diagnosis of the classic appearing of Stafne cyst, in the posterior lingual aspect of the mandible, is easily made with a panoramic radiograph. Stafne cyst's differential diagnosis includes odontogenic or non-odontogenic cystic lesions, fibrous dysplasia, non-ossifying fibroma, brown tumor of hyperparathyroidism, osteoporotic bone marrow defect, ameloblastoma, giant cell tumor, benign salivary gland tumors, hemangioma, myxoma, multiple myeloma, eosinophilic granuloma and metastatic disease.^9^ Also considering the mass filling the cavity, benign or malignant lymphadenopathy, lymphomas, cystic hygroma, hemangiomas, neurogenic tumors, benign or malign glandular tumors should be in mind.^10^ With the use of Computerized Tomography (CT), CT-sialography and Magnetic Resonance Imaging (MRI), differential diagnosis can be made.

All authors made an agreement that Stafne bone cyst does not require surgical treatment and radiologic follow-up should be recommended for the management of the bone depression, because it can be considered an anatomical condition rather than a pathological entity. Biopsy or surgical exploration should be thought only if the diagnosis is uncertain or unusual exceptional disease is suspected. In the present case we preferred surgical exploration based on MRI findings presenting with anatomic deficiency. Histopathologic outcome revealed a developmental pathology, ectopic parotid gland which according to our knowledge is the first case in the English literature.

## Conclusion

Stafne bone cyst is a rare, benign anatomic entity mostly seen as posterior lingual depression of mandible and in our case it is associated with aberrant but well organized parotid glandular tissue originating dislocation from the pharyngeal pouches. According to our case and previous reported cases, advanced imaging studies must be obtained in order to eliminate the possibility of potentially dangerous diseases.

## Conflicts of interest

The authors declare no conflicts of interest.
